# A study on performance evaluation of surgical departments in tertiary public hospitals based on the fuzzy combination-DEA method

**DOI:** 10.3389/fpubh.2026.1801363

**Published:** 2026-04-15

**Authors:** Shuting Wu, Yongzhe Chen, Yixin Wu, Xiongwei Wu

**Affiliations:** Fuzhou University Affiliated Provincial Hospital, Fuzhou, China

**Keywords:** Boston matrix, DEA (data envelopment analysis), fuzzy combination, health policy, performance, surgical department

## Abstract

**Background:**

Tertiary grade-A public hospitals serve as key concentrations of high-quality medical resources in China. However, the allocation of resources within these hospitals remains a concern, with surgical departments facing particular challenges in both service capacity and operational efficiency, potentially hindering the provision of high-quality care. Therefore, this study aims to explore an appropriate comprehensive evaluation method for assessing the operational efficiency and service capacity of surgical departments in public hospitals, thereby providing a decision-making basis for specialty development.

**Methods:**

Based on key performance indicators for public hospitals, the service capacity of surgical departments in a tertiary hospital was evaluated using the entropy-weighted TOPSIS method combined with the Rank Sum Ratio (RSR) method through fuzzy integration, while operational efficiency was assessed using Data Envelopment Analysis (DEA). A comprehensive analysis of departmental effectiveness was conducted using the Boston Matrix.

**Results:**

In terms of medical service capacity evaluation, five ranking results were obtained using the fuzzy integration method. The top three departments were consistently A, B, and C, while the bottom two were consistently R and S. Regarding operational efficiency evaluation, the overall operational efficiency of the 19 surgical departments was 0.835. Nine departments were DEA-efficient, while ten were DEA-inefficient. Comprehensive analysis using the Boston Matrix revealed: Departments A, B, D, and I were high-efficiency, high-capability departments, demonstrating strong medical service capacity and high operational efficiency. Departments G, K, L, P, Q, R, and S were high-efficiency, low-capability departments, exhibiting relatively high operational efficiency but insufficient medical service capacity. Departments C, E, and F were low-efficiency, high-capability departments, with strong medical service capacity but inadequate operational efficiency. Departments H, J, M, N, and O were low-efficiency, low-capability departments, showing both insufficient medical service capacity and low operational efficiency.

**Conclusion:**

The evaluation results obtained from the entropy-weighted TOPSIS method and the fuzzy combination of the Rank-Sum Ratio (RSR) method demonstrated a high degree of consistency. When combined with DEA-based efficiency assessment, these methods enable a more scientific and precise evaluation of surgical department effectiveness from two distinct dimensions, thereby providing a robust decision-making basis. Surgical departments in the sample hospital should prioritize technological advancement and the optimization of talent structure, shifting the focus of resource allocation from “incremental expansion” to “structural optimization”.

## Background

In China, public hospitals constitute a vital component of the healthcare system, undertaking the principal healthcare services. Deepening the implementation of the High-Quality Development Initiative and the Public Hospital Performance Assessment are core tasks as outlined in the Key Tasks for Deepening the Medical and Healthcare System Reform in 2024 (1). Against the backdrop of the ongoing DRG-based medical insurance payment system reform, public hospitals are confronting substantial operational pressures and challenges (2, 3). A central challenge for their high-quality development is how to effectively integrate performance assessment indicators into routine departmental management, leveraging evaluation to drive disciplinary development, enhance medical quality, optimize operational efficiency, and promote refined departmental management.

In China, Diagnosis-Related Groups (DRG) have been widely applied as a risk adjustment tool in the field of health management. Core DRG indicators have also been incorporated into the national performance evaluation of tertiary public hospitals and the accreditation standards for tertiary hospitals. Currently, many hospitals have integrated DRG metrics, such as the Case Mix Index (CMI), into the performance assessments of departments and physicians. This approach leverages performance evaluation to guide clinical practices, actively encouraging departments to admit patients with complex and critical conditions. Such a strategy not only enhances the hospital’s economic performance under the context of DRG-based payment by medical insurance but also aligns with the social functional orientation expected of tertiary hospitals. It adheres to relevant national policy requirements under the new healthcare reform, thereby promoting the high-quality development of hospitals (4–6).

As a crucial component of hospital advancement, surgical departments are of paramount importance. Conducting scientific evaluation and in-depth analysis of their operational data facilitates the identification of challenges in specialty development, which is essential for enhancing the overall level and comprehensive strength of a hospital (7). Current research primarily focuses on constructing indicator systems or employing single-method evaluations. For instance, Bootstrap Data Envelopment Analysis (Bootstrap-DEA) (8), a novel additive star-NDEA model (9), and traditional DEA models (10) have been utilized to calculate operational efficiency scores.

**Figure 1:**
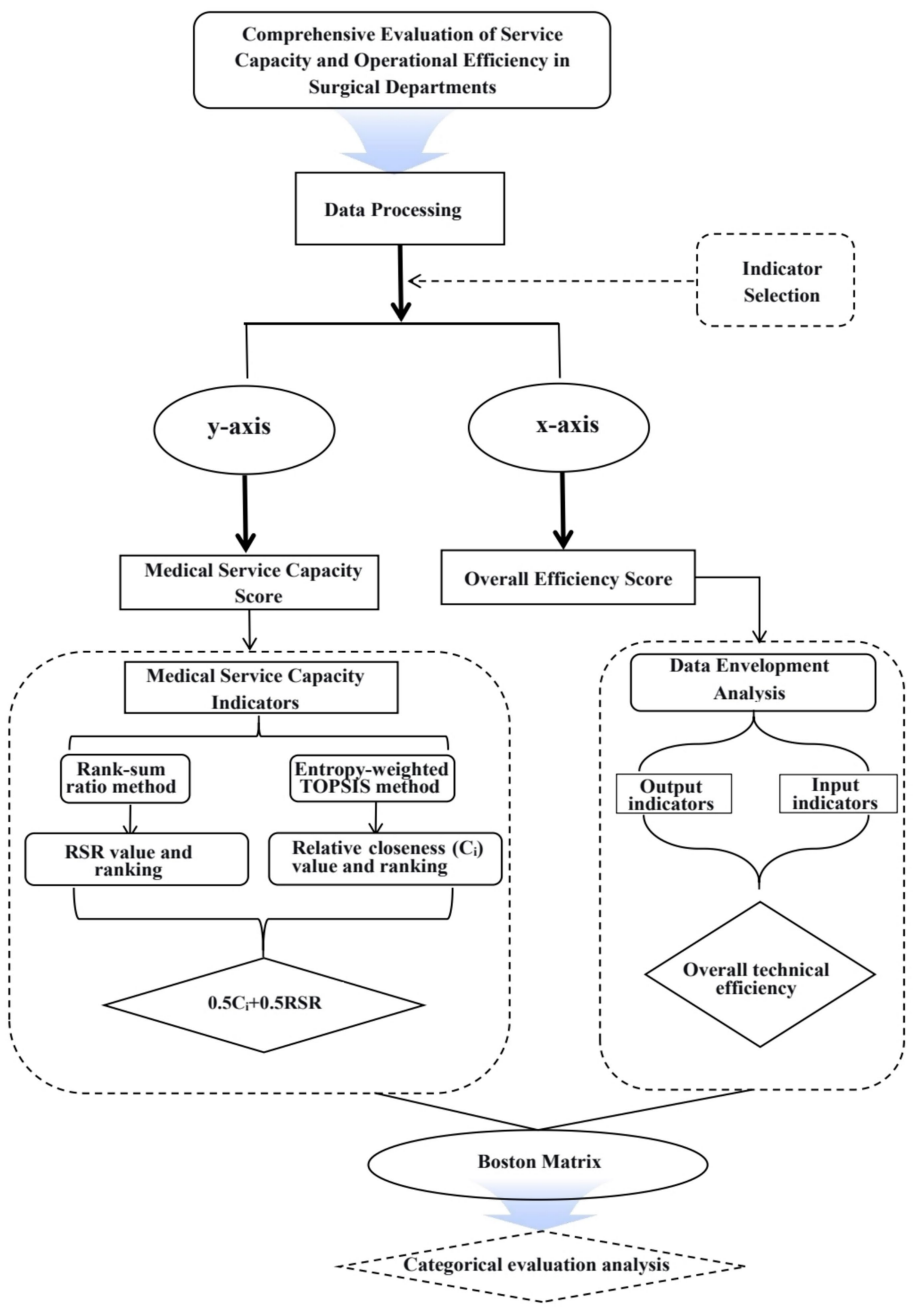
Analytical framework diagram.

This study, taking the surgical departments of a tertiary Grade-A hospital as an example, constructs a comprehensive performance evaluation framework. Drawing on key performance indicators for public hospitals, the framework integrates the entropy-weighted TOPSIS method with the Rank-Sum Ratio (RSR) method via fuzzy combination evaluation (11), and employs Data Envelopment Analysis (DEA). By mapping the results onto a Boston Matrix along the two dimensions of medical service capacity and operational efficiency, this approach enables a more scientific, precise, and intuitive visualization of the comprehensive effectiveness of surgical departments, thereby providing decision-making support for their high-quality development (Figure 1).

## Materials and methods

### Data sources

This study investigated 19 surgical departments in a tertiary grade-A public hospital in 2024. The hospital, a comprehensive facility located in Fujian Province, China, has 3,005 designated beds and handles approximately 2.8 million outpatient and emergency visits, 125,000 discharges, and 60,000 inpatient surgical procedures annually. It comprises 36 clinical departments, among which 19 perform surgical operations and were all enrolled in this study. These departments encompass the following specialties: Thoracic Surgery, Gastrointestinal Surgery, Hepatopancreatobiliary Surgery, Neurosurgery, Department of Cardiovascular Surgery (Division I), Department of Cardiovascular Surgery (Division II), Urology, Department of Orthopedics (Division I), Department of Orthopedics (Division II), Gynecology, Ophthalmology, Thyroid and Hernia Surgery, Emergency Surgery, Pediatric Surgery, Breast Surgery, Vascular and Interventional Oncology, Otolaryngology, Obstetrics, and Plastic Surgery and Burn Unit (Represented by the letters A-S).

Data were sourced from the hospital’s internal National Public Hospital Performance Evaluation Indicator Management System, medical record management system, and human resources reports. The data were processed using Microsoft Excel and DEAP2.1 software.

### Research methods

#### Indicator selection

Drawing on the Notice on the Issuance of the Assessment Method for National Clinical Specialty Capacity (Trial) and the Notice on the Issuance of the Evaluation Indicators for the High-Quality Development of Public Hospitals (Trial) issued by the General Office of the National Health Commission, and informed by a comprehensive literature review, this study selected seven indicators to evaluate medical service capacity. The selection process adhered to the principles of representativeness, data availability, and standardized measurement, while also taking into account the specific operational characteristics of surgical departments. The seven indicators are: Case Mix Index (CMI, where a higher value indicates greater complexity of diseases treated), average length of stay, number of discharged patients, proportion of level-4 surgeries, proportion of minimally invasive surgeries, number of cases with RW ≥ 2 (a metric used to quantify the technical difficulty of cases, with RW ≥ 2 typically representing highly complex and resource-intensive surgical procedures), and inpatient medical service revenue. Among these, CMI and RW are explicitly designated in the Assessment Method for National Clinical Specialty Capacity (Trial) as evaluation indicators reflecting technical capability. Average length of stay serves as an indicator of service efficiency. The proportion of level-4 surgeries, the proportion of minimally invasive surgeries, and inpatient medical service revenue are all indicators associated with the “structural optimization” dimension outlined in the Evaluation Indicators for the High-Quality Development of Public Hospitals (Trial). Furthermore, these seven indicators constitute key monitoring metrics within the DRG-based payment reform framework. Accordingly, the selection of these seven indicators as the evaluation metrics for medical service capacity in this study is both well-grounded and methodologically justified (12, 13).

To ensure consistency in the evaluation, the aforementioned seven indicators were simultaneously employed as output indicators in the Data Envelopment Analysis (DEA). Among these, CMI, the number of cases with RW ≥ 2, the proportion of level-4 surgeries, and the proportion of minimally invasive surgeries primarily reflect the technical complexity of diagnoses and treatments, the severity of case mix, and the level of medical technology (14). The number of discharged patients and inpatient medical service revenue mainly capture the scale and output level of medical services. The reciprocal of average length of stay was used to reflect the efficiency of bed utilization and hospital operations. Collectively, these indicators represent the hospital’s medical service output across three dimensions-technical complexity, service scale, and operational efficiency-thereby providing a relatively comprehensive reflection of the core elements underpinning high-quality hospital development.

Because average length of stay is a negative indicator (lower values indicate better performance), its reciprocal was used in the DEA model to align with the positive orientation of other output indicators. Although some degree of inherent correlation exists among certain indicators, they represent distinct dimensions of output and hold synergistic significance in hospital operations. Therefore, including them together in the output indicator system facilitates a comprehensive and objective evaluation of hospital efficiency. For this study, two indicators-the number of physicians and the number of actual open beds-were selected as input indicators for the DEA. Due to constraints in data availability, other resources (such as operating room utilization time and medical equipment) were not included as input indicators.

### Fuzzy integration of the TOPSIS and RSR methods

The entropy weight method is a multi-criteria decision analysis technique employed to address decision-making problems involving multiple indicators or criteria (15). Grounded in the concept of information entropy, this method determines the weight of each indicator by quantifying the amount of useful information embedded in the data, thereby reflecting their relative importance in the decision-making process in an objective manner (16, 17). The specific formulas are presented as follows (18):

1. Equations 1, 2 were used to normalize beneficial and non-beneficial indicators, respectively.

$$X_{\mathrm{ij}}^{\prime }=\frac{X_{\mathrm{ij}}-\min(X_{\mathrm{ij}})}{\max(X_{\mathrm{ij}})-\min(X_{\mathrm{ij}})}\;\;X_{\mathrm{ij}}^{\prime }=\frac{\max(X_{\mathrm{ij}})-(X_{\mathrm{ij}})}{\max(X_{\mathrm{ij}})-\min(X_{\mathrm{ij}})}$$ (1)

2. The information entropy and information were calculated according to the constructed decision matrix Value of utility. The entropy is the Equation 2. The weight is the Equation 3.

$$p_{\mathrm{ij}}=\frac{x_{\mathrm{ij}}}{\sum_{i=1}^{n}x_{\mathrm{ij}}}\;\;k=\frac{1}{\ln(m)}\;\;E_{j}=-k\sum_{i=1}^{n}p_{\mathrm{ij}}\times \ln(p_{\mathrm{ij}})$$ (2)

$$w_{\mathrm{ij}}=\frac{1-E_{j}}{n-\sum_{i=1}^{n}E_{j}}$$ (3)

The TOPSIS method identifies the optimal and worst-ideal solutions among all alternatives based on a normalized initial data matrix. By calculating the distance between each evaluation object and these ideal solutions, it assesses the relative closeness to the optimal solution, which serves as the basis for performance evaluation (19, 20).

The entropy-weighted TOPSIS method employs the entropy weight method to calculate the weight of each evaluation indicator. These weights are then multiplied by the corresponding indicator values to obtain weighted values, after which the TOPSIS method is applied to determine the ranking of the evaluation objects, thereby providing a more objective assessment. An entropy-weighted standardized matrix was constructed from the raw data to serve as the foundational dataset for the TOPSIS method (21). The positive and negative ideal solutions were calculated separately for positive and negative indicators, followed by the computation of the weighted Euclidean distances (D_i_^+^ and D_i_^−^). Subsequently, the relative closeness (C_i_), where a larger C_i_ value indicates better service capacity of the hospital. The specific formulas are as follows:

$$\begin{array}{l} D_{i}^{+}=\sqrt{\left(\sum_{i=1}^{m}w_{i}{\left(Z_{\mathrm{ij}}-Z_{\mathrm{ij}}^{+}\right)}^{2}\right)}\;\;D_{i}^{-}=\sqrt{\left(\sum_{i=1}^{m}w_{i}{\left(Z_{\mathrm{ij}}-Z_{\mathrm{ij}}^{-}\right)}^{2}\right)}\;\;\\ \;\;\;\;\;\;\;\;\;\;\;\;\;\;\;\;\;\;\;\;\;\;\;\;\;\;\;\;\;\;\;\;\;\;\;\;\;\;\;\;\;\;\;\;\;\;\;\;\;\;\;\;\;\;\;\;\;\;\;\;\;\;\;\;\;\;\;\;\;\;\;\;\;\;\;\;\;\;C_{i}=(D_{i}^{-})/(D_{i}^{+}+D_{i}^{-})\times 1 \end{array}$$

The Rank-Sum Ratio (RSR) method involves converting raw data within an n × m matrix into a dimensionless statistic, RSR, through rank transformation. The RSR values are then arranged in ascending order to assign group ranks (R), followed by calculation of the cumulative frequency (P). The NORMSINV function is used to convert the cumulative frequency P into a probability unit (Probit), enabling the ranking or graded classification of evaluation objects. This method is suitable for comprehensive evaluations involving indicators with different units of measurement (22).

The fuzzy integration of the TOPSIS and RSR methods is a comprehensive evaluation approach that integrates the two individual evaluation methods based on fuzzy set theory, thereby leveraging the advantages of both. This integration overcomes the limitations of a single method in addressing uncertainty, efficiency, and preference trade-offs, thereby enhancing the accuracy and sensitivity of the evaluation results. The integration is achieved through the formula W₁Cᵢ + W₂RSR, where Cᵢ and RSR represent the results from the TOPSIS and RSR methods, respectively. In this study, sensitivity analysis was conducted by varying the weight ratios assigned to the two methods in the fuzzy combination. The weight ratio W1: W2 are applied as 1:0, 0.9:0.1, 0.5:0.5, 0.9:0.1, 0:1, respectively (18, 20).

### Data envelopment analysis (DEA)

Data Envelopment Analysis (DEA) is a widely employed method for efficiency evaluation. It utilizes linear programming to measure the relative efficiency of decision-making units (DMUs) in transforming multiple inputs into multiple outputs. In addition, DEA analyzes input and output slacks to identify the magnitude and direction of improvements required for inefficient DMUs to reach the efficiency frontier (23–25).

Traditional Data Envelopment Analysis (DEA) models can be categorized into the CCR model and the BCC model. The CCR model calculates overall technical efficiency under the assumption of constant returns to scale, while the BCC model, assuming variable returns to scale, decomposes overall technical efficiency into pure technical efficiency and scale efficiency, thereby facilitating the identification of sources of inefficiency (26). For inefficient decision-making units, the BCC model not only identifies the directions for indicator adjustment but also provides specific adjustment magnitudes. Input slack represents the maximum reducible amount of a particular input resource while maintaining the current output level, whereas output shortfall denotes the maximum expandable amount of a specific output while holding the current input level constant. Therefore, employing the input-oriented BCC model enables not only the measurement of operational efficiency but also a deeper exploration of the underlying causes of inefficiency. This approach offers valuable insights for decision-making and aligns more closely with the practical context of uneven resource allocation and varying developmental levels across departments. Furthermore, given that hospital departments are not profit-driven and exercise greater managerial control over input factors such as medical staff and beds, an input-oriented model was adopted. This orientation aims to provide a basis for optimizing resource input and enhancing operational efficiency (27).

This study employed the BCC model as the analytical framework for efficiency evaluation to calculate the comprehensive efficiency of each clinical department. A comprehensive efficiency score equal to 1 indicates that the decision-making unit (DMU) is DEA-efficient, reflecting a balanced input–output relationship. A score below 1 denotes DEA-inefficiency, signifying an imbalance between inputs and outputs. Comprehensive efficiency is derived as the product of pure technical efficiency and scale efficiency, representing the overall performance of a DMU in terms of both technological and scale-related factors (23, 24).

## Results

### Evaluation results of the entropy-weighted TOPSIS method

The entropy weight method was employed to calculate the entropy values and entropy weights of the indicators used to evaluate the medical service capacity of surgical departments (Table 1). Among these indicators, the number of cases with RW ≥ 2 exhibited the highest entropy weight (0.2993) and the greatest degree of variation, indicating that increasing the volume of high-weight complex cases significantly contributes to enhancing the medical service capacity of hospitals. This finding reflects the hospital’s strategic focus on actively targeting complex and critical cases and strengthening its capacity to manage such conditions—a direction aligned with the objectives of DRG-based payment reform. Moreover, it is consistent with the national policy orientation for public hospitals, which encourages “enhancing the medical service capacity for critical cases” and promotes “a transition from scale expansion toward improvements in quality, efficiency, and technology.” This result is in accordance with findings from related studies (7, 28).

**Table 1:** Entropy values and entropy weights of the evaluation indicators for medical service capability in surgical departments.

Indicator	CMI	Average length of stay	Number of discharges	Proportion of Level-IV surgeries	Proportion of minimally invasive surgeries	Number of cases with RW ≥ 2	Inpatient medical service revenue
Information entropy	0.9223	0.9651	0.8716	0.8641	0.8413	0.7149	0.8681
Weight	0.0816	0.0366	0.1348	0.1426	0.1666	0.2993	0.1385

The results indicated that average length of stay exhibited the lowest entropy weight (0.0366) and the smallest degree of variation among all indicators. This finding suggests that, as a traditional core indicator of hospital operational efficiency, average length of stay has entered a plateau and a steady-state range following years of refined management and performance appraisal guidance, with relatively limited potential for further improvement. As a result, the differences between departments are not significant, leading to its relatively low informational content and discriminatory power. Consequently, this indicator contributed a smaller weight in the comprehensive evaluation.

Based on the weights of each evaluation indicator calculated by the entropy weight method, the relative closeness (C_i_) and ranking of each surgical department were derived through the TOPSIS method. A C_i_ value closer to 1 indicates stronger medical service capability in that surgical department, whereas a value closer to 0 reflects weaker capacity (38). The results, presented in Table 2, showed that C_i_ values ranged from 0.0441 to 0.9251, revealing a significant imbalance and marked disparity in the development of medical service capacity across different surgical departments. Specifically, the Obstetrics department (R), Plastic Surgery and Burn Unit (S), Breast Surgery department (P), and Otolaryngology department (N) exhibited relatively low C_i_ values and ranked in the lower tier, indicating comparatively weaker medical service capacity.

**Table 2:** Evaluation results of the entropy-weighted TOPSIS method for medical service capability in surgical departments.

Department	D^+^	D^−^	*C_i_*	Ranking
A	0.0085	0.1047	0.9251	1
B	0.0398	0.0693	0.6355	2
C	0.0675	0.0439	0.3939	3
F	0.0698	0.0386	0.3560	4
I	0.0800	0.0298	0.2712	5
E	0.0880	0.0304	0.2566	6
D	0.0897	0.0219	0.1961	7
K	0.0882	0.0194	0.1807	8
G	0.0926	0.0203	0.1797	9
H	0.0983	0.0201	0.1695	10
L	0.1021	0.0199	0.1635	11
J	0.0997	0.0159	0.1375	12
O	0.0958	0.0122	0.1132	13
M	0.1003	0.0121	0.1080	14
Q	0.1035	0.0119	0.1031	15
P	0.1009	0.0087	0.0797	16
N	0.1029	0.0086	0.0774	17
R	0.1045	0.0048	0.0442	18
S	0.1038	0.0048	0.0441	19

Analysis based on departmental attributes and operational characteristics revealed that the low C_i_ values observed in these four departments are attributable to distinct specialty-specific features. These departments primarily manage standardized cases, such as natural deliveries and cesarean sections, perform less technically demanding surgeries (e.g., breast surgeries and management of mild-to-moderate burns), and implement day surgery pathways. The cases they handle typically involve shorter disease courses, relatively lower Resource Weight (RW) values, and a lower Case Mix Index (CMI), while their bed turnover rate is comparatively faster and resource consumption per admission is relatively concentrated. The low proportion of complex and critical cases places these departments at a disadvantage within an evaluation system that assigns higher weight to high-difficulty, high-complexity, and high-RW cases. Furthermore, in assessments emphasizing volume-based indicators such as the number of discharged patients and inpatient medical service revenue, these departments are also often at a disadvantage. Consequently, their comprehensive scores remain at a relatively low level.

### Evaluation results of the rank sum ratio (RSR) method

The Rank-Sum Ratio (RSR) values for each surgical department were calculated, sorted in ascending order to assign ranks (R), and subsequently used to compute the downward cumulative frequency (P) and the corresponding probit values. A higher RSR value indicates stronger medical service capacity in the respective surgical department.

The results (Table 3) showed that the RSR values ranged from 0.2256 to 0.8421, indicating substantial variation across departments. The top three surgical departments in terms of medical service capacity were A, B, and C, which was consistent with the rankings obtained from the entropy-weighted TOPSIS method. The bottom three departments were Q, R, and S, showing slight variation compared to the rankings derived from the entropy-weighted TOPSIS method.

**Table 3:** Evaluation results of medical service capability in surgical departments using the RSR method.

Department	RSR value	*P*	Probit value	RSR ranking
A	0.8421	0.9868^*^	7.2203	1
B	0.8120	0.9474	6.6199	2
C	0.7331	0.8947	6.2521	3
D	0.6541	0.8421	6.0031	4
E	0.6466	0.7632	5.7165	5
F	0.6466	0.7632	5.7165	5
G	0.5677	0.6842	5.4795	7
H	0.5639	0.6316	5.3360	8
I	0.5564	0.5789	5.1992	9
J	0.5113	0.5263	5.0660	10
K	0.4662	0.4662	4.8008	11
L	0.4662	0.4662	4.8008	11
M	0.4361	0.3421	4.5933	13
N	0.4361	0.3421	4.5933	13
O	0.4060	0.2632	4.3664	15
P	0.3985	0.2105	4.1954	16
Q	0.3684	0.1579	3.9969	17
R	0.2632	0.1053	3.7479	18
S	0.2256	0.0526	3.3801	19

* Indicates correction by [1 − (1/4n)] × 100%.

### Results of the fuzzy joint evaluation

This study integrated the entropy-weighted TOPSIS method and the Rank Sum Ratio (RSR) method to conduct a fuzzy comprehensive evaluation of the assessment results. The findings (Table 4) indicate that the top three ranked departments (A, B, and C) and the bottom two departments (S and R) were completely consistent across all fuzzy combination schemes.

**Table 4:** Fuzzy joint ranking based on the entropy-weighted TOPSIS and RSR methods.

Department	Entropy-weighted TOPSIS method		RSR method		Fuzzy comprehensive evaluation					
	C_i_	Ranking	RSR	Ranking	0.1C_i_ + 0.9RSR	Ranking	0.5C_i_ + 0.5RSR	Ranking	0.9C_i_ + 0.1RSR	Ranking
A	0.9251	1	0.8421	1	0.8504	1	0.8836	1	0.9168	1
B	0.6355	2	0.8120	2	0.7944	2	0.7238	2	0.6531	2
C	0.3939	3	0.7331	3	0.6992	3	0.5635	3	0.4278	3
D	0.1961	7	0.6541	4	0.6083	5	0.4251	6	0.2419	7
E	0.2566	6	0.6466	5	0.6076	6	0.4516	5	0.2956	6
F	0.3560	4	0.6466	5	0.6176	4	0.5013	4	0.3850	4
G	0.1797	9	0.5677	7	0.5289	7	0.3737	8	0.2185	8
H	0.1695	10	0.5639	8	0.5245	9	0.3667	9	0.2090	10
I	0.2712	5	0.5564	9	0.5279	8	0.4138	7	0.2997	5
J	0.1375	12	0.5113	10	0.4739	10	0.3244	10	0.1749	12
K	0.1807	8	0.4662	11	0.4376	11	0.3234	11	0.2092	9
L	0.1635	11	0.4662	11	0.4359	12	0.3148	12	0.1937	11
M	0.1080	14	0.4361	13	0.4033	13	0.2720	13	0.1408	14
N	0.0774	17	0.4361	13	0.4002	14	0.2568	15	0.1133	16
O	0.1132	13	0.4060	15	0.3767	15	0.2596	14	0.1425	13
P	0.0797	16	0.3985	16	0.3666	16	0.2391	16	0.1116	17
Q	0.1031	15	0.3684	17	0.3419	17	0.2358	17	0.1297	15
R	0.0442	18	0.2632	18	0.2413	18	0.1537	18	0.0661	18
S	0.0441	19	0.2256	19	0.2074	19	0.1348	19	0.0622	19

The overall consistency of the ranking results derived from the five evaluation methods was assessed using Cronbach’s *α* coefficient and the intraclass correlation coefficient (ICC). The results showed (Table 5): Cronbach’s α = 0.994, average measure ICC = 0.994 (95% CI: 0.989–0.998, *p* < 0.001), and the Spearman correlation coefficients among the methods ranged from 0.936 to 0.996 (*p* < 0.001). These findings indicate a strong and statistically robust overall consistency among the rankings produced by the five analytical approaches, thereby confirming the stability and reliability of the evaluation results.

**Table 5:** Spearman correlation matrix between different ranking methods (*N* = 19).

Method	C_i_	RSR	0.1C_i_ + 0.9RSR	0.5C_i_ + 0.5RSR	0.9C_i_ + 0.1RSR
C_i_	1	0.936	0.956	0.972	0.996
RSR	0.936	1	0.994	0.986	0.949
0.1C_i+_0.9RSR	0.956	0.994	1	0.995	0.967
0.5C_i_ + 0.5RSR	0.972	0.986	0.995	1	0.979
0.9C_i_ + 0.1RSR	0.996	0.949	0.967	0.979	1

Considering the complementary advantages of the methods, the robustness of the results, and established academic conventions (18, 20), an equal-weight combination (0.5:0.5) was ultimately selected as the final score for departmental medical service capacity. This combined approach effectively integrates the strengths of both the TOPSIS and RSR evaluation methods, attenuates the potential bias inherent in any single method, and yields a more objective and reliable evaluation outcome.

### Evaluation of operational efficiency in surgical departments based on data envelopment analysis (DEA)

This study employed the same set of 19 surgical departments as decision-making units (DMUs) for both the Data Envelopment Analysis (DEA) and the entropy-weighted TOPSIS and RSR evaluations. Furthermore, the input–output indicator system used in the DEA was consistent with the comprehensive evaluation indicators in terms of definition, data source, and time period, ensuring the comparability, coherence, and robustness of the multi-method joint evaluation. During the data preprocessing stage, all input and output indicators were verified. No negative values were present; however, the proportion of minimally invasive surgeries and the number of cases with RW ≥ 2 had isolated zero values in one department (Ophthalmology), reflecting its actual operational circumstances. To meet the computational requirements of DEA, these zero values were uniformly adjusted by adding 0.01. This adjustment does not alter the relative differences among departments nor does it affect the evaluation results.

A multiple linear regression model was constructed with average length of stay as the dependent variable, and CMI, number of discharged patients, proportion of level-4 surgeries, proportion of minimally invasive surgeries, number of cases with RW ≥ 2, and inpatient medical service revenue as independent variables. Multicollinearity among the independent variables was assessed using the variance inflation factor (VIF) and tolerance. The results showed that the VIF values for the variables ranged from 3.006 to 6.729, and tolerance values ranged from 0.149 to 0.333. All values met the conventional statistical thresholds (VIF < 10 and tolerance > 0.1), indicating the absence of severe multicollinearity in the regression model.

The input and output data of the 19 surgical departments were incorporated into an input-oriented BCC model under the assumption of variable returns to scale to measure their operational efficiency. The results (Table 6) show that the average comprehensive efficiency, pure technical efficiency, and scale efficiency of the surgical departments were 0.835, 0.876, and 0.954, respectively. Among the 19 departments, 9 exhibited constant returns to scale (DEA effective), 7 exhibited increasing returns to scale, and 3 exhibited decreasing returns to scale (the Departments of Gastrointestinal Surgery, Hepatopancreatobiliary Surgery, and Urology). Among these, Departments C and E exhibit “Decreasing Returns to Scale.” However, the table shows C with scale efficiency 0.953 and E with 0.588. Departments with decreasing returns to scale have exceeded their optimal operational scale; reducing input scale could improve efficiency. This finding suggests that where resource allocation has surpassed the optimal production scale, further expansion would not improve efficiency but would instead diminish overall operational performance.

**Table 6:** Evaluation results of operational efficiency in surgical departments.

Department	Comprehensive efficiency	Pure technical efficiency	Scale efficiency	Returns to scale
A	1.000	1.000	1.000	Constant returns to scale
B	0.839	1.000	0.839	Decreasing returns to scale
C	0.707	0.742	0.953	Decreasing returns to scale
F	0.569	0.571	0.996	Increasing returns to scale
I	1.000	1.000	1.000	Constant returns to scale
E	0.588	1.000	0.588	Decreasing returns to scale
D	0.976	0.992	0.984	Increasing returns to scale
K	1.000	1.000	1.000	Constant returns to scale
G	1.000	1.000	1.000	Constant returns to scale
H	0.537	0.557	0.965	Increasing returns to scale
L	1.000	1.000	1.000	Constant returns to scale
J	0.665	0.687	0.967	Increasing returns to scale
O	0.610	0.661	0.923	Increasing returns to scale
M	0.735	0.801	0.917	Increasing returns to scale
Q	1.000	1.000	1.000	Constant returns to scale
P	1.000	1.000	1.000	Constant returns to scale
N	0.630	0.631	0.998	Increasing returns to scale
R	1.000	1.000	1.000	Constant returns to scale
S	1.000	1.000	1.000	Constant returns to scale
Mean	0.835	0.876	0.954	

The mean comprehensive efficiency in this study was 0.835, which falls within the range commonly reported in efficiency studies of departments in tertiary grade-A public hospitals in China (7, 21, 29–32), indicating relatively sound overall performance while highlighting room for further optimization. For departments identified as DEA-inefficient, further analysis was conducted to examine their input slacks and output shortfalls (Table 7).

**Table 7:** Input redundancy and output shortfall in surgical departments.

Department	Redundancy in input indicators		Deficiency in output indicators						
	Number of physicians (persons)	Number of actually open beds (units)	CMI	Average length of stay (days)	Number of discharges (cases)	Proportion of level-4 surgeries (%)	Proportion of minimally invasive surgeries (%)	RW ≥ 2 (cases)	Medical service revenue (10,000 CNY)
A	0.0	0.0	0.0	0.0	0.0	0.0	0.0	0.0	0.0
B	0.0	0.0	0.0	0.0	0.0	0.0	0.0	0.0	0.0
C	0.0	19.8	0.1	0.0	0.0	37.4	0.0	1268.2	486.1
F	0.0	0.0	0.0	0.0	0.0	0.0	17.7	195.6	292.9
I	0.0	0.0	0.0	0.0	0.0	0.0	0.0	0.0	0.0
E	0.0	0.0	0.0	0.0	0.0	0.0	0.0	0.0	0.0
D	0.0	0.0	0.0	0.0	1484.2	0.0	33.4	1186.3	1249.6
K	0.0	0.0	0.0	0.0	0.0	0.0	0.0	0.0	0.0
G	0.0	0.0	0.0	0.0	0.0	0.0	0.0	0.0	0.0
H	0.0	0.0	0.5	0.0	0.0	13.9	0.0	1387.5	596.3
L	0.0	0.0	0.0	0.0	0.0	0.0	0.0	0.0	0.0
J	0.0	0.0	0.7	0.0	0.0	0.0	16.9	1264.1	408.2
O	0.0	0.0	0.0	0.1	855.8	0.0	26.9	582.8	959.3
M	0.0	10.6	0.3	0.0	0.0	21.0	0.0	793.5	738.3
Q	0.0	0.0	0.0	0.0	0.0	0.0	0.0	0.0	0.0
P	0.0	0.0	0.0	0.0	0.0	0.0	0.0	0.0	0.0
N	0.0	0.0	0.0	0.2	0.0	0.0	11.9	52.9	281.9
R	0.0	0.0	0.0	0.0	0.0	0.0	0.0	0.0	0.0
S	0.0	0.0	0.0	0.0	0.0	0.0	0.0	0.0	0.0

The results revealed that departments C and M exhibited bed redundancies of 19.8 and 10.6, respectively. Adjusting the number of beds could therefore enhance their operational efficiency. Furthermore, under current input levels, Departments C, F, D, H, J, O, M, and N all demonstrated varying degrees of output shortfalls. This finding indicates that, while resource input levels are relatively adequate, deficiencies persist in operational efficiency and structural optimization. Therefore, management efforts should prioritize process optimization, case-mix adjustment, technological capability enhancement, and efficiency improvement. Certain departments (D and O) exhibited substantial shortfalls in the number of discharged patients, representing theoretical annual improvement targets projected by the DEA model. While this reflects significant potential for efficiency enhancement in these departments, it should be regarded as a reference for guiding managerial improvement rather than as an absolute target that must be fully achieved in practice.

### Boston matrix analysis based on the fuzzy combination-DEA method

**Figure 2:**
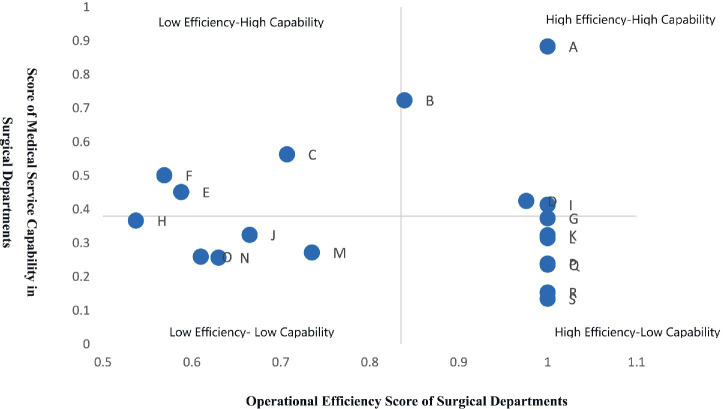
Boston matrix of medical service capability and operational efficiency in surgical departments.

Based on the aforementioned evaluation results, a Boston Matrix was constructed by taking the medical service capacity score of surgical departments, derived from the fuzzy combination method (0.5Cᵢ + 0.5RSR), as the vertical axis and the operational efficiency value, measured by the DEA method, as the horizontal axis. The mean overall technical efficiency was used as the cutoff point for the efficiency dimension, and the mean medical service capacity score served as the cutoff point for the capacity dimension, thereby classifying the 19 surgical departments into four categories (Figure 2): High Efficiency-High Capability, High Efficiency-Low Capability, Low Efficiency-High Capability, and Low Efficiency-Low Capability. Departments classified as High Efficiency-High Capability exhibited both strong medical service capacity and high operational efficiency, namely A, B, D, and I, accounting for 21.05% of the departments. Those in the High Efficiency-Low Capability category demonstrated relatively weak medical service capacity but high operational efficiency, specifically G, K, L, P, Q, R, and S, representing 36.84% of the total. The Low Efficiency-High Capability type included departments with strong medical service capacity yet poor operational efficiency, namely C, E, and F, comprising 15.79%. Finally, departments classified as Low Efficiency-Low Capability showed both weak medical service capacity and low operational efficiency, specifically H, O, N, J, and M, accounting for 26.32% of the departments.

In this study, departments classified as low efficiency but high capacity accounted for 36.84% of the total, representing the largest proportion among all categories. This phenomenon may reflect the inherent specialization and division of labor within the hospital setting.

## Discussion

### Scientific analysis of the comprehensive evaluation methods

In performance evaluation, a single evaluation method has inherent limitations and cannot simultaneously address “efficiency, preferences, uncertainty, and multi-dimensional trade-offs.” However, existing literature has documented the application of the entropy-weighted TOPSIS method and the combined TOPSIS-RSR method. These approaches have been widely employed in retrospective comparative assessments of the overall performance of healthcare institutions across different regions, comparisons between various medical facilities, evaluations across different departments within the same hospital, and assessments of comprehensive capacity among different wards within the same clinical department (32, 33). The innovation of this study lies in the fuzzy integration of the entropy-weighted TOPSIS method and the RSR method, which effectively reduces subjective intervention in determining the weights and setting scoring rules of each indicator (14). The rankings of departments derived from different evaluation methods showed a consistent overall trend, aligning with actual conditions. More importantly, by incorporating the comprehensive efficiency measured by the DEA method, this study conducted a comprehensive evaluation across two independent dimensions—medical service capacity and operational efficiency—and presented the findings using the Boston Matrix. This approach enables a more scientific and precise analysis of the performance of surgical departments.

### Strategies for high-quality development of surgical departments

Based on the distinct characteristics of different surgical departments, hospitals should formulate differentiated development strategies to enhance specialty-specific capabilities and operational efficiency (29, 34).

For departments characterized by both high efficiency and high capability (A, B, D, and I), it is advisable to designate them as key disciplines for high-quality development. Thoracic Surgery Department (A), Gastrointestinal Surgery Department (B), Orthopedics Department II (D) and Cardiovascular Surgery Department II (I) should consider establishing specialized disease diagnosis and treatment centers, enhancing the integration of surgical and non-surgical specialties, prioritizing diseases with high Case Mix Index (CMI) values, concentrating efforts on breakthroughs in cutting-edge technologies, and striving to strengthen their regional influence.

Departments exhibiting lower efficiency but relatively strong service capacity (C, E, and F) possess a solid foundation for enhancing value output under the DRG payment model. However, notable deficiencies persist in their resource allocation and operational management. Taking Department C (Hepatobiliary and Pancreatic Surgery) as an example, evident bed redundancy suggests that its bed scale has exceeded the optimal output level, exhibiting characteristics of diseconomies of scale. In the context of DRG-driven cost containment and resource constraints, redundant beds should be appropriately reduced and reallocated. The freed-up bed resources should be prioritized for departments demonstrating increasing returns to scale, high CMI, and high output efficiency, thereby enhancing the overall value contribution of hospital-wide resources. Department F (Neurosurgery) exhibits a shortfall in minimally invasive surgery output, reflecting a misalignment between its current technical structure and the developmental direction of high-value-added services under DRG.

Departments classified as high-efficiency but low-capability accounted for 36.84% of the total, representing the largest proportion among all categories. These departments include G (First Department of Orthopedics), K (First Department of Cardiovascular Surgery), L (Ophthalmology), P (Vascular and Tumor Interventional Radiology), Q (Breast Surgery), R (Obstetrics), and S (Plastic and Burn Surgery). This finding indicates that although these departments demonstrate streamlined operational processes, adequate resource utilization, and relatively effective cost control, their overall performance is constrained by factors such as disciplinary focus, case mix, technical complexity, and disease severity. As a result, they exhibit relative deficiencies in CMI, the proportion of level-4 surgeries, and the number of cases with RW ≥ 2, leading to comparatively low comprehensive service capacity. This phenomenon objectively reflects the inherent specialization and division of labor within the hospital. Nevertheless, these departments retain substantial development potential and should pursue growth through differentiated competition, overcoming capability bottlenecks by adopting minimally invasive and specialty-specific techniques to achieve intrinsic disciplinary enhancement. The hospital should strategically strengthen training in minimally invasive procedures, reinforce the development of the talent echelon, and upgrade minimally invasive equipment to address current technical limitations. Such efforts would increase both the volume and technical sophistication of minimally invasive surgeries, thereby enabling higher RW output and improved operational efficiency with equivalent resource inputs.

For departments with low levels of both efficiency and capacity, relying solely on internal optimization efforts is insufficient to quickly adapt to the requirements of DRG-based development. Innovative development approaches and cross-departmental collaboration mechanisms should be adopted. First, establish surgical collaboration and specialty co-development mechanisms, whereby advantageous departments provide technical guidance, joint surgeries, and expert support to rapidly enhance the complex case management capabilities of weaker departments. Second, promote disciplinary integration and service line reorganization by consolidating low-volume departments with similar case types, implementing a model of integrated specialties with focused subspecialties, and concentrating resources to achieve excellence in priority disease groups. Third, adopt differentiated positioning and case-mix optimization, guiding departments to focus on appropriate technologies and regionally essential disease types, thereby avoiding low-level homogeneous competition and progressively improving CMI and RW structure. Fourth, leverage hospital-level platforms to implement standardized processes and cost control, utilizing shared platforms for anesthesia, surgery, critical care, and DRG cost management to reduce operational costs and enhance operational efficiency.

### Pathways to high-quality hospital development under the diagnosis-related group (DRG) payment model

Under the DRG payment system, hospital revenue is no longer determined solely by discharge volume and operational scale, but is heavily dependent on the Case Mix Index (CMI), disease weight (RW), technical complexity, and resource consumption structure (35). Complex and critical cases correspond to higher RW values and reimbursement rates, serving as core drivers of long-term revenue growth and disciplinary competitiveness. In contrast, low-weight routine cases, while operationally efficient, offer limited profit margins and insufficient growth potential. Consequently, the prevalence of numerous departments operating in a state of high efficiency but low capacity represents a structural risk that warrants attention. Such departments may sustain short-term operational efficiency but are likely to struggle in supporting the hospital’s value enhancement and sustainable development under DRG. Prolonged absence of technological upgrading and structural optimization will gradually lead to stagnant revenue growth, declining resource contribution, weakened disciplinary competitiveness, and may even suppress the hospital’s overall CMI level, ultimately undermining long-term returns and developmental momentum.

Consequently, under the DRG paradigm, hospitals cannot solely pursue operational efficiency but must also prioritize value output, technological advancement, and structural optimization (4, 30). For high-efficiency, low-capacity departments, the management focus should progressively shift from cost control and turnover enhancement toward strengthening technological capacity, optimizing case structure, and increasing case weight. Through targeted initiatives in talent cultivation, equipment allocation, technical support, and case-mix guidance, their capacity to admit high-RW complex cases and perform level-4 surgeries should be enhanced. This would facilitate the transformation of these departments from “high efficiency, low value” toward “high efficiency, high value” star departments. Only by achieving simultaneous improvements in both efficiency and capacity, along with synergistic development of scale and value, can hospitals sustain stable long-term revenue and achieve high-quality development in the context of DRG payment reform.

This study has several limitations. First, the analysis was based on data from a single hospital. The unique positioning, resource allocation, patient population, and management model of this specific institution constrain the generalizability of the findings, limiting their applicability to other hospitals of different levels or in different regions. Second, this study employed cross-sectional data from a single time point in 2024. This approach provides only a static snapshot for that specific period and fails to capture dynamic trends in the study variables over time. It also cannot rule out the confounding effects of short-term fluctuations on the results. Third, this study did not include indicators related to medical quality and safety, such as complication rates and mortality rates. Medical quality and safety constitute the fundamental benchmarks of healthcare services; therefore, evaluating the actual effectiveness of medical care without such indicators remains incomplete. Fourth, potential endogeneity issues may exist in the Data Envelopment Analysis (DEA) process. Relying solely on the Case Mix Index (CMI) may not fully capture the true heterogeneity of the case mix. Factors such as individual patient differences and comorbidities were not adequately considered, which could potentially introduce biases into the DEA efficiency evaluation results. Future research should develop hierarchical and categorized management evaluation methods, dynamically adjust the combination of evaluation indicators based on different decision-making needs and orientations, and apply various evaluation results to management decisions such as disciplinary development, performance appraisal, and resource allocation, thereby promoting refined and high-quality development in hospitals.

## Conclusion

When formulating disciplinary development strategies, hospitals should not only consider department types but also comprehensively integrate various subjective and objective factors to develop targeted incentive policies (36, 37). Emphasis should be placed on technological advancement and the optimization of talent structure, establishing a hospital-wide dynamic bed allocation mechanism (i.e., “one bed for the whole hospital”) and implementing digital and intelligent shared management models. Additionally, strengthening the construction of medical alliances and optimizing the case mix are essential.

## Data Availability

The original contributions presented in the study are included in the article/supplementary material, further inquiries can be directed to the corresponding author.
